# Porphyrin Photoabsorption and Fluorescence Variation with Adsorptive Loading on Gold Nanoparticles

**DOI:** 10.3389/fchem.2021.777041

**Published:** 2021-11-23

**Authors:** Akira Shinohara, Guang Shao, Takashi Nakanishi, Hideyuki Shinmori

**Affiliations:** ^1^ School of Chemistry, Sun Yat-sen University, Guangzhou, China; ^2^ Faculty of Life and Environmental Science, Graduate Faculty of Interdisciplinary Research, Department of Biotechnology, University of Yamanashi, Kofu, Japan; ^3^ International Center for Materials Nanoarchitectonics (WPI-MANA), National Institute for Materials Science (NIMS), Tsukuba, Japan

**Keywords:** porphyrin, gold nanoparticle, photoabsorption, fluorescence, adsorbate

## Abstract

Here, we report the photophysical structure–property relationship of porphyrins adsorbed on gold nanoparticles. The number of porphyrin–alkanethiolate adsorbates per particle was adjusted by a post-synthetic thiol/thiolate exchange reaction on 1-dodecanethiolate–protected gold nanoparticles. Even with a low loading level of adsorbates (<10% of all thiolate sites on gold nanoparticles), the shoulder absorption at the Soret band was intensified, indicating the formation of aggregates of porphyrin adsorbates on the nanoparticles. Steady-state fluorescence quantum yields could be adjusted by the bulkiness of substituents at the *meso*-positions of the porphyrin or the methylene linker chain length, regardless of the porphyrin loading level and the nanoparticle diameter.

## 1 Introduction

The design of dye molecules with specific photophysical properties is necessary for many applications including optoelectronics, photocatalysis, and bioimaging (Lu and Nakanishi, 2019; Watanabe et al., 2019; Sato, 2020; Han et al., 2021). Various approaches to customize the properties of such dyes have been explored, based on the thorough investigation of the photophysical structure–property relationship (Shin and Lee, 2002; Benniston et al., 2003; Kovaliov et al., 2014; Kulinich et al., 2016). Despite these efforts, fine-tuning the properties that are intrinsic to the electronic structure of dyes remains challenging because the molecular design approach enables only stepwise adjustments.

One of the alternative approaches to adjusting the photophysical properties is to conjugate dyes and metal nanoparticles (Dulkeith et al., 2002; Doering and Nie, 2003; Pan et al., 2006; Hasobe, 2021). Nanoparticles are usually defined as particles with a diameter between ∼1 and ∼100 nm (Kreibig and Vollmer, 1995). In this size range, the chemical, photophysical, and electrical properties differ dramatically in comparison with the bulk counterparts (Glotov et al., 2019). For practical applications, gold is one of the few appropriate candidates, due to its chemical inertness. Gold nanoparticles (AuNPs), often protected with alkanethiolate groups as developed by Brust *et al.*(Brust et al., 1994) in the past quarter of a century, are widely employed for this purpose because of their extraordinary redispersibility (Thomas and Kamat, 2003). The alkanethiolate adsorbates can readily be replaced with other thiolates by thiol/thiolate exchange reactions to obtain AuNPs modified by incoming adsorbates. Additional desired functional properties such as the solubility, charge, and affinity with other molecules can be added or amended by successive or coincident adsorbate exchange reactions to afford multifunctional nanoparticles (Ingram et al., 1997).

Porphyrins and their analogs have potential applications in many fields—sensing, catalysis, and photovoltaics, for example—so have attracted interest in their unique photophysics, including huge absorption coefficients of ∼5 × 10^5^ M^−1^ cm^−1^ (Kubo et al., 2003; Kinoshita et al., 2012; Barona–Castaño et al., 2016; Ghosh et al., 2019). Thorough investigations on the photophysics of porphyrin–AuNP conjugates have been conducted. For instance, Akiyama *et al.* reported photocurrent enhancement in porphyrin–AuNP conjugates by a localized surface plasmon resonance (LSPR)–enhanced electromagnetic field (Akiyama et al., 2006). Imahori *et al.* reported the fluorescence lifetime of porphyrin adsorbates on AuNPs was significantly longer than those on a two-dimensional Au substrate (Imahori et al., 2001). The general strategy for the adjustment of photophysical properties of dye–AuNP conjugates involves altering the size and shape of the nanoparticles and the length of the linkers (Imahori et al., 1998; Dulkeith et al., 2005; Hong and Li, 2013; Do and Imae, 2021). However, less attention has been paid to the number of adsorbates loaded per particle, despite the strong influence on photophysical properties (Shinohara and Shinmori, 2016).

Here, we present the photophysical properties of porphyrin adsorbates on AuNPs having different loading levels, substituents on the *meso*-positions of porphyrin, lengths of alkanethiolate linkers, and nanoparticle diameters. Porphyrin–AuNP conjugates, with the adsorbate loading levels of up to 10% of all available thiolate sites, were obtained by post-synthetic thiol/thiolate exchange reactions employing 1-dodecanethiolate–protected AuNPs as the precursors. By characterizing conjugates with various loading levels, we have identified the factors that determine the photoabsorption and fluorescence properties of porphyrin adsorbates on the AuNPs.

## 2 Results and Discussion

### 2.1 Synthesis of Porphyrin–Gold Nanoparticle Conjugates

**FIGURE 1:**
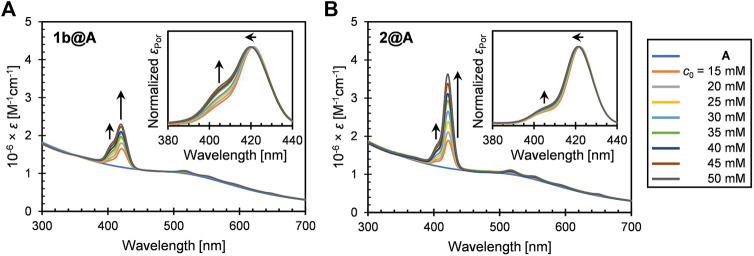
UV-vis extinction spectra (in toluene) of **(A)** 1b@A and **(B)** 2@A prepared by post-synthetic modification of nanoparticle **A** with different initial concentrations of porphyrin–alkanethiols (*c*_0_). Insets represent the normalized absorption spectra of porphyrin adsorbates.

**SCHEME 1:**
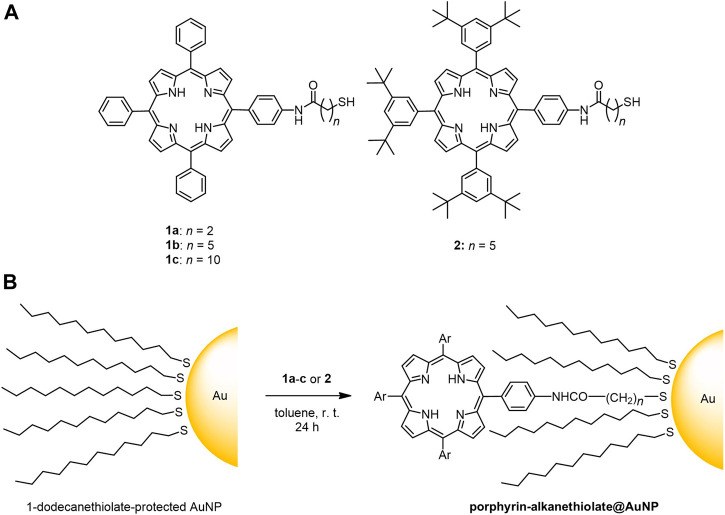
**(A)** Chemical structure of porphyrin–alkanethiols 1a-c and 2; **(B)** post-synthetic loading of porphyrin–alkanethiols on 1-dodecanethiolate-protected AuNPs.

**SCHEME 2:**
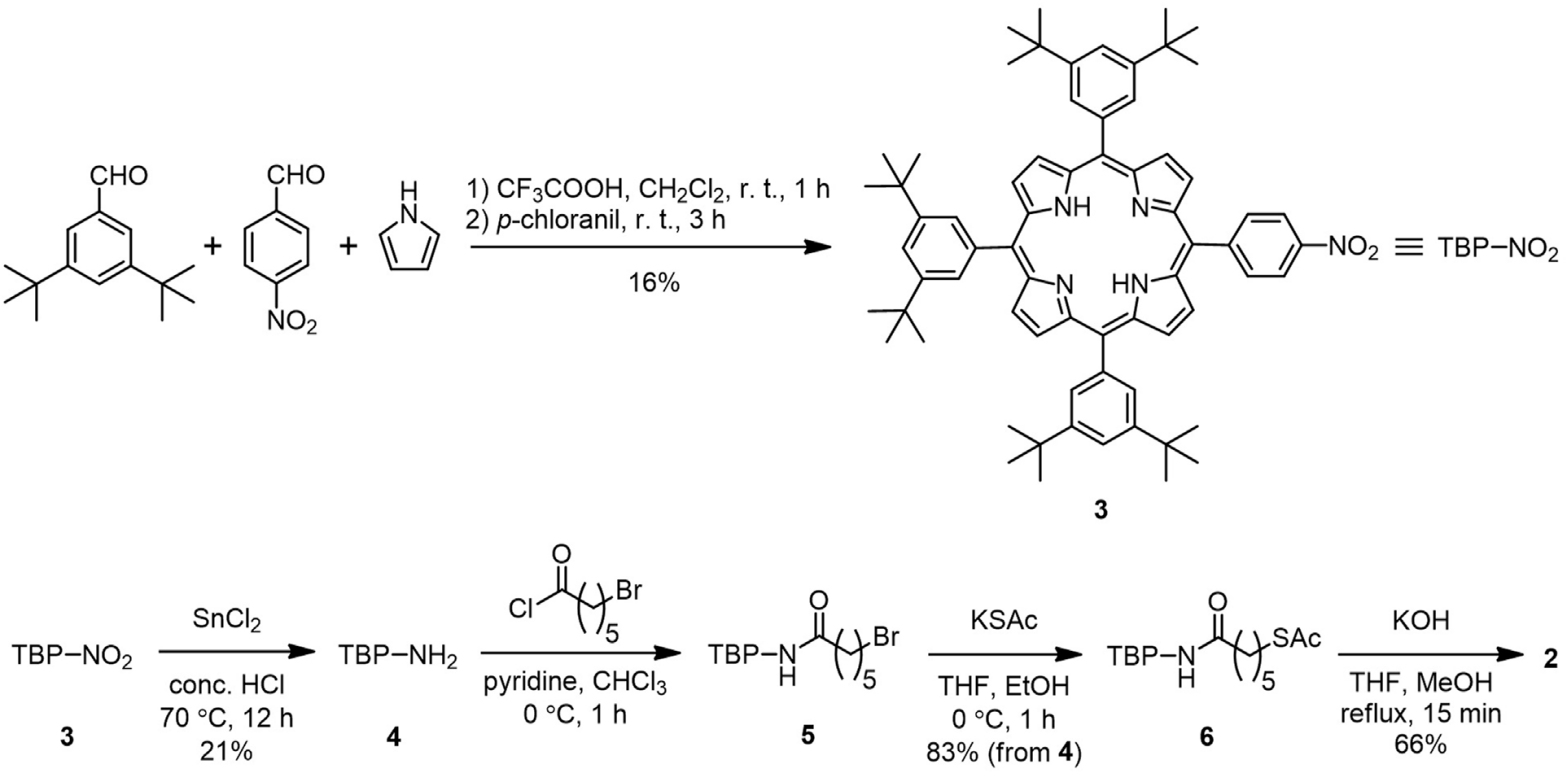
Synthetic route to porphyrin–alkanethiol 2 with bulky *tert*-butyl substituents.

We employed three types of 1-dodecanethiolate–protected AuNPs A–C which have different diameters and distributions (A: 2.5 ± 0.5, B: 2.5 ± 0.9, and C: 5.6 ± 2.2 nm). The 2.5-nm species (A and B) showed weak LSPR around 510 nm (Figure 1), while the resonance was distinct for the 5.6-nm species C at 516 nm (Jin, 2010). Four porphyrin–alkanethiols, three with different methylene linker chain lengths (1a–c, *n* = 2, 5, and 10) and one with bulkier substituents at three *meso*-positions of porphyrin (2, *n* = 5) were chosen as adsorbates (Scheme 1). Compound 2 was synthesized using a route similar to those of 1a–c, as previously reported (Scheme 2) (Shinohara and Shinmori, 2016).

The post-synthetic modification of 1-dodecanethiolate–protected AuNPs, namely, thiol/thiolate exchange reaction, was performed by mixing with porphyrin–alkanethiols in toluene, followed by purification by gel permeation chromatography to remove unreacted porphyrin–alkanethiols. Hereafter, the porphyrin–AuNP conjugates are denoted as porphyrin–alkanethiolate@AuNPs. 1a–c@B and 1a–c@C that we previously prepared (Shinohara and Shinmori, 2016) were used for the photophysical characterization of the conjugates. The procedure of the thiol/thiolate exchange reaction was slightly modified for the synthesis of 1b@A and 2@A, where the initial concentrations of porphyrin–alkanethiols were adjusted (*c*_0_ = 15–50 mM) under a constant concentration of the AuNPs, and the reaction time extended to obtain samples with different loading levels. The range of loaded porphyrin–alkanethiolate adsorbates per particle (*N*) were determined to be 1.2–3.1 (1b@A) and 1.5–5.5 (2@A) of 126 total thiolate sites (Terrill et al., 1995), respectively. Detailed procedures for the synthesis of A, 2, 1b@A, and 2@A and the determination of *N* are given in the Experimental Section.

### 2.2 UV-Vis Extinction Spectra

The UV-vis extinction spectra of 1b@A and 2@A (*n* = 5, 2.5 ± 0.5 nm) in toluene are shown in Figure 1. Both the conjugates exhibit the weak LSPR band of the AuNPs around 510 nm, B band (Soret band) around 420 nm, and four Q bands in the range of 500–700 nm. The spectra indicate the successful loading of porphyrin–alkanethiolates on the AuNPs. The five absorption maxima of porphyrin adsorbates were red-shifted up to 4 nm (Supplementary Tables S1, S2) compared with those of the corresponding porphyrin–alkanethiols (1b and 2, Supplementary Figure S1). A similar bathochromic effect is also observed in other dye–nanoparticle conjugates (Prasanna et al., 2014; Ashjari et al., 2015).

While the extinction spectra of porphyrin–alkanethiols for 1b and 2 are almost identical (Supplementary Figure S1), the absorption features of porphyrin adsorbates on the AuNP show different trends. It should be noted that shoulder growth on the shorter wavelength side of the Soret band around 405 nm was observed, which could be consistent with the blue-shifted absorption band arising from *H*-aggregate exciton formation (Eisfeld and Briggs, 2006). The vertical molecular orientation of the porphyrins on the AuNPs through Au–S covalent bonds allows face-to-face aggregations between neighboring porphyrin adsorbates. This is in contrast to the edge-to-edge *J*-aggregation of the porphyrin adsorbates on AuNPs using multivalent linking, where red-shifted shoulder growth is observed (Kanehara et al., 2008; Ohyama et al., 2009). No observable shoulder growth was found in the Q bands, as predicted by Kasha’s theory that chromophores with a higher molar absorption coefficient show stronger exciton couplings (Kasha, 1976).

**FIGURE 2:**
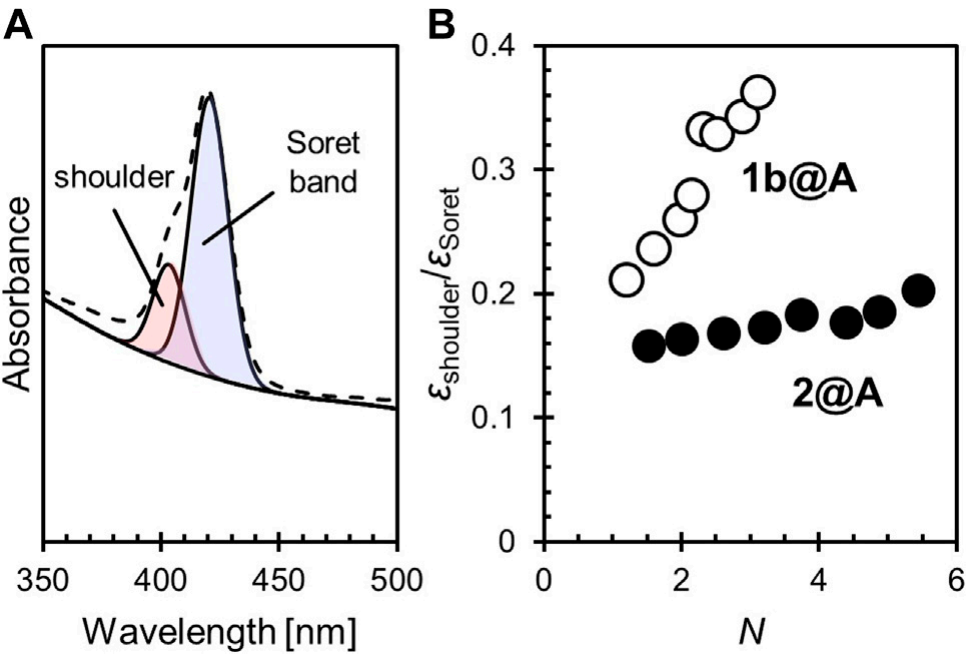
**(A)** Spectral deconvolution of the Soret band and shoulder into two Gaussian curves. **(B)** The relative shoulder intensity ( $\varepsilon_{\text{shoulder}}/\varepsilon_{\text{Soret}}$ ) of porphyrin adsorbates with different numbers of porphyrin–alkanethiolate adsorbates per particle (*N*).

We qualified the aggregation of porphyrin adsorbates on AuNPs by the ratio of the molar absorption coefficients of the shoulder (∼405 nm) to the Soret band (∼420 nm), $\varepsilon_{\text{shoulder}}/\varepsilon_{\text{Soret}}$ (Figure 2A, Supplementary Tables S3, S4). The shoulder growth of 1b@A with increasing *N* strongly indicates the formation of face-to-face *H*-aggregates (Figure 2B). A significantly slower shoulder growth rate of 2@A likely results from the steric effect of the bulky aryl groups at *meso*-positions. The aryl groups that substitute the *meso*-positions of the porphyrin have a large dihedral angle (60–90°) from the porphyrin π plane (Rayati et al., 2008). Therefore, the introduction of the bulky *tert*-butyl groups at the 3 and 5 positions of the aryl groups increase their face-to-face distances in the *H*-aggregates and therefore weaken the exciton coupling (Imahori et al., 2004a).

In these conjugates, only a small fraction of the 1-dodecanethiolate sites were exchanged for porphyrin–alkanethiolate (see 2.1). It may seem strange that the porphyrin adsorbates form the aggregates on the AuNPs, despite their low loading level. It is known that the Au–S covalent adsorbates on the AuNPs dynamically migrate. This enables the self-assembly of adsorbates on the AuNPs when the formation of aggregates is thermodynamically favored (Werts et al., 2004; Tanaka et al., 2006). The aggregation tendency of porphyrins by π–π stacking may contribute to the self-assembly of porphyrins on the AuNPs. Also, the amide bond that links the porphyrin moiety is another functional group that can contribute to the aggregation *via* hydrogen bonds. Such an electrostatic interaction would provide a dominant cohesive force compared with π–π stacking in the nonpolar environment (i.e., in toluene) (Pérez–Rentero et al., 2014) and so help one porphyrin adsorbate access an adjacent porphyrin adsorbate followed by aggregation (Sanz et al., 2012).

The broadening of the Soret band (i.e., the increase in the full width at half maximum (FWHM)) was also remarkable in 1b@A but not in 2@A. Interestingly, unlike the shoulder growth, which depends on *N*, the FWHM only shows a slight expansion according to the increase in *N* (1b: 12.2 nm, 2: 11.3 nm, 1b@A: 17.0–18.5 nm, and 2@A: 14.2–15.1 nm, Supplementary Tables S3, S4). It has been shown, in other systems, that the broadening of the absorption band, which is also observed in chromophores adsorbed on two-dimensional Au substrates, arises from interactions between chromophores and other molecules (e.g., other adsorbates or solvent) and is independent of exciton coupling (Leontidis et al., 1995). The porphyrin adsorbates can interact with not only the AuNPs and other porphyrins but also the remaining 1-dodecanethiolate adsorbates. Although the formation of porphyrin aggregates seems to result in this broadening, in the case of 1b@A, the effect of loading level (i.e., the number of possible porphyrin–porphyrin interactions) is much smaller (18.5–17.0 = 1.5 nm) than other effects (17.0–12.2 = 4.8 nm) associated with the porphyrin loading onto the AuNPs. The smaller broadening for 2@A (15.1–14.2 = 0.9 nm of the effect of a loading level, and 14.2–11.3 = 2.9 nm of other effects) indicates that the bulky *tert*-butyl substituents inhibit not only porphyrin–porphyrin interactions but also interactions with other molecules (e.g., 1-dodecanethiolate adsorbates).

### 2.3 Steady-State Fluorescence Spectra and Fluorescence Quantum Yields

**FIGURE 3:**
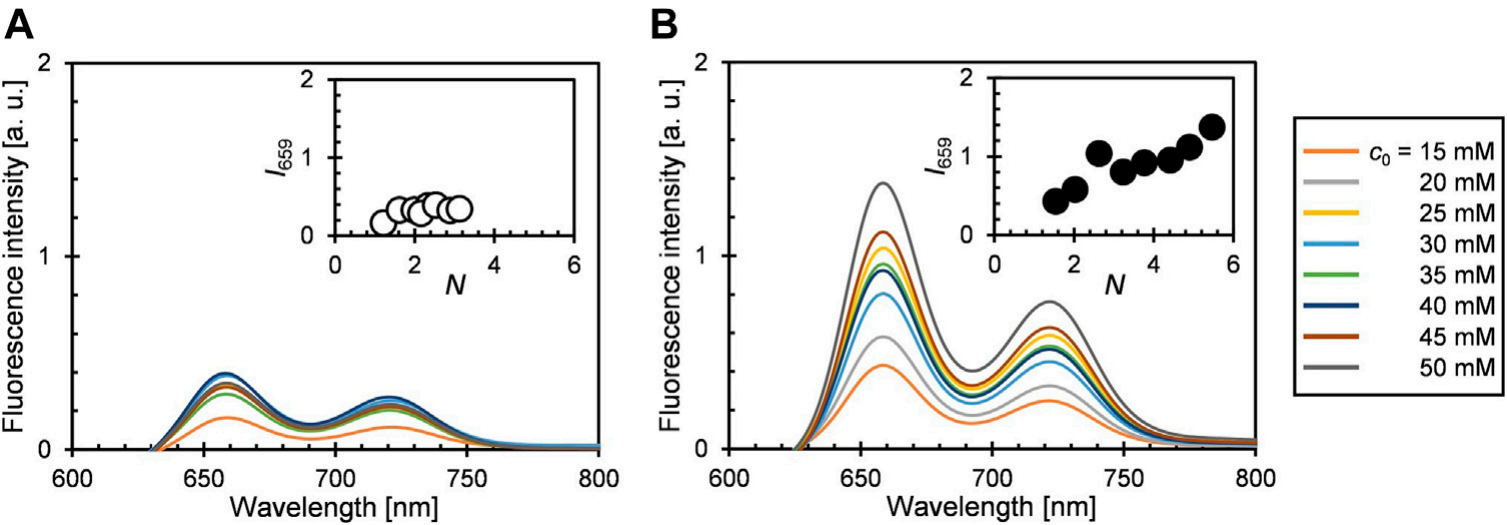
Steady-state fluorescence spectra of **(A)** 1b@A and **(B)** 2@A in toluene (saturated with air at *p*O_2_ = *ca.* 0.21 atm, *λ*_ex_ = 515 nm). Insets show fluorescence intensity at 659 nm (*I*_659_, Q_(0–0)_ band) versus *N*.

**FIGURE 4:**
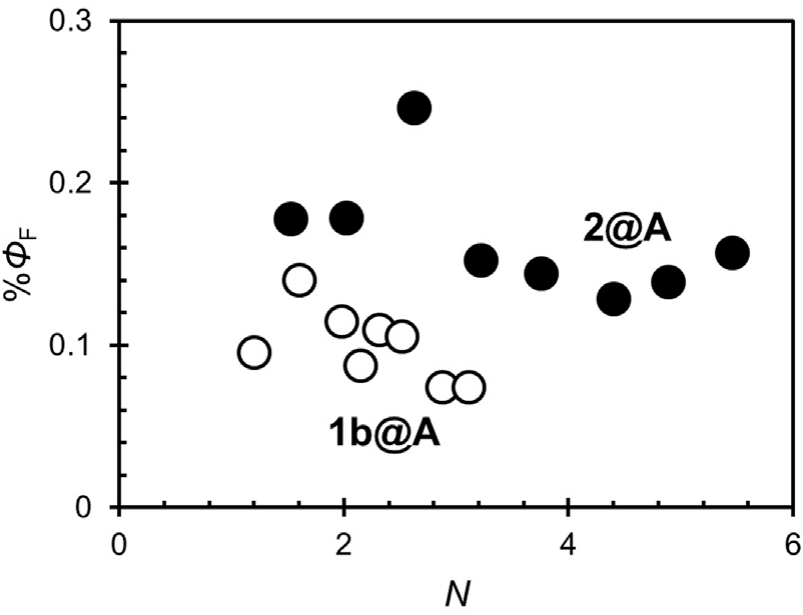
Fluorescence quantum yields (*Φ*_F_) of 1b@A and 2@A with different *N*.

The steady-state fluorescence spectra of 1b@A and 2@A with various *N* are shown in Figure 3. Two characteristic emission bands were observed at 658 nm and 721–722 nm, respectively (Supplementary Tables S1, S2) (Akimoto et al., 1999). Emission maxima (*λ*_em_) were slightly red-shifted on loading (*λ*_em_: 1b: 654 and 720 nm and 2: 655 and 721 nm) but not affected by *N*, which is similar to the tendency of the absorption spectra. The fluorescence of the porphyrin adsorbates on the AuNPs is almost quenched (fluorescence quantum yields *Φ*_F_ = 0.07–0.14% (1b@A), 0.13–0.25% (2@A), Figure 4) compared to the absence of AuNPs (*Φ*_F_ = 11% (1b), 12% (2)). Such strong quenching of the fluorescence by the AuNPs is broadly found in various fluorophores such as pyrene, anthracene, and rhodamine 6G (Uznański et al., 2009; Kabb et al., 2015; Shaikh et al., 2015).

It is known that most fluorophores are non-fluorescent in *H*-aggregates, in contrast to *J*-aggregates which exhibit fluorescence (Liu et al., 2015). In present conjugates, excited porphyrin adsorbates can be quenched not only by the AuNPs but also by the adjacent porphyrin adsorbates. However, *Φ*_F_ is nearly independent of *N* (Figure 4), unlike the singlet oxygen quantum yields (*Φ*_Δ_), which decreases exponentially as *N* increases (Shinohara and Shinmori, 2016). This fact indicates that the porphyrin–porphyrin energy transfer is not the dominant pathway for the quenching of fluorescence in the conjugates. Statistical analysis showed *Φ*_F_ of 2@A (0.15 ± 0.02) is higher than that of 1b@A (0.10 ± 0.02%) upon removing the outlier at *N* = 2.6 that fell more than two standard deviations (SD) above the mean (Table 1; the results of statistical rejection and significance tests are shown in Supplementary Tables S5, S6). Another potential factor that influences quench efficiency is the spatial freedom of the adsorbates. In this regard, bulky substituents inhibit aggregation and increase the spatial degree of freedom of porphyrin fluorophore, resulting in reduced energy transfer efficiency from porphyrin adsorbates to AuNPs (Best et al., 2007).

**TABLE 1:** Average fluorescence quantum yields (*Φ*_F_) of porphyrin adsorbates on AuNPs with different diameters and size distributions.

Adsorbate	*Φ*_F_ *^a^* on AuNP [%]		
	A*^b^* (2.5 ± 0.5 nm)	B*^c^* (2.5 ± 0.9 nm)	C*^c^* (5.6 ± 2.2 nm)
1a	———	0.11 ± 0.04	0.14 ± 0.03
1b	0.10 ± 0.02	0.13 ± 0.02	0.15 ± 0.01
1c	———	0.20 ± 0.02	0.19 ± 0.01
2	0.15 ± 0.02 (0.17 ± 0.03*^d^*)	———	———

a In toluene, saturated with air at *p*O_2_ = *ca*. 0.21 atm.

Sample count.

b *n* = 8.

c *n* = 5.

d Value before the outlier point *N* = 2.6; %*Φ*_F_, 0.25 was rejected.

### 2.4 Effects of Linker Chain Length and Nanoparticle Size on Fluorescence

The porphyrin–AuNP conjugates 1a–c@B and 1a–c@C were synthesized as described and determined to have *N* ranges 3–15 (126 total thiolate sites) and 9–43 (632 total thiolate sites), respectively (spectral and statistical data are shown in Supplementary Figures S3–S6 and Supplementary Tables S6–S19). The *ε*_shoulder_/*ε*_Soret_ increases with *N* on both nanoparticles but shows a non-monotonic trend with the length of the side chain (1b > 1c > 1a, Supplementary Figure S4A,B). As mentioned before, the space around the porphyrin becomes larger with the lengthening of the linker and smaller with the tendency of the adsorbate to aggregate. Based on this idea, the aggregation should be the strongest for 1a (*n* = 2), but it was not in the results. Steric repulsion between the adsorbents is expected to be one of the factors that determine the aggregation formation in the successive adsorptive loading. One particular consideration in the adsorptive loading onto the nanoparticles to form three-dimensional monolayers is the curvature of the substrate surface. At the same curvature, the shorter linkers increase the porphyrin–porphyrin steric repulsion, which is thought to inhibit the formation of aggregates (Terrill et al., 1995) (Supplementary Figure S7A,B). The steric hindrance may also reduce the thiolate density on the gold surface, making such monolayers thermodynamically unfavorable (Ingram et al., 1997). The universal trend in the shoulder growth in adsorbates 1b (*n* = 5) and 1c (*n* = 10), which is independent of the particle size, was found (Supplementary Figure S4C). This fact supports our hypothesis: in the adsorptive loading, porphyrins are preferentially loaded next to the already introduced adsorbates and form aggregates. On the other hand, in 1a (*n* = 2), the universal trend was no longer held. This deviation suggests that the aggregation in 1a on the larger nanoparticles C, due to the π–π stacking and/or the hydrogen bonds between the amide groups, with a small curvature is thermodynamically unfavorable (Supplementary Figure S7C,D). Imahori et al.(Imahori et al., 2000) reported that for tetraarylporphyrin–amide–alkanethiolate (the same structure as the present system), the porphyrin plane is oriented perpendicular to the two-dimensional Au substrate surface when *n* is an odd number, which is preferable to form *H*-aggregates (Supplementary Figure S8A). When *n* is an even number, the porphyrin plane is tilted toward the substrate surface, resulting in weaker exciton coupling by *H*-aggregation (Supplementary Figure S8B). The competitive effects on the aggregation strength are considered to result in the non-monotonic tendency.

The average *Φ*_F_ of the conjugates is shown in Table 1. In both series of conjugates, *Φ*_F_ slightly increased with methylene linker chain length (*n* = 2, 5, and 10). Note that no significant difference was found between *n* = 2 and 5 (Supplementary Table S6), which is considered to be due to the large variance of *n* = 2 (SD = 0.03–0.04%). Strong aggregation at *n* = 5 (odd number) is likely to contribute to the reduction in the fluorescence quantum yield, however is not, as evidenced by the lack of dependence on *N*. This is consistent with previous reports of an increasing donor–acceptor distance reducing quenching efficiency (Rao and Mayor, 2005; Deng et al., 2019). In addition, similar to the effect of bulky substituents, lengthening of the linker chain increases the space around the end groups (i.e., porphyrin adsorbates) and may inhibit the quenching of fluorescence (Badia et al., 1996). There are numerous reports on LSPR-induced fluorescence enhancement, as well as the enhancement of photoabsorption, Raman scattering (Chung et al., 2011; Bauch et al., 2014; Zvyagina et al., 2018), and singlet oxygen generation (Shinohara and Shinmori, 2016); however, no statistically significant difference was found between the LSPR-silent (**1a**–**c@B**) and LSPR-active (**1a**–**c@C**) conjugates (Supplementary Table S6). This can be attributed to the fact that the quenching process, which depends on the porphyrin–nanoparticle distance, is dominant in the range of linker chain lengths (*n* = 2–10).

## 3 Experimental Section

### 3.1 General

All starting materials and reagents were purchased from commercial suppliers and used without further purification. Spectroscopic-grade toluene was saturated with air (partial pressure of oxygen *p*O_2_ = *ca.* 0.21 atm) before use.

### 3.2 Apparatus

UV–vis spectra were recorded on a Shimadzu UV-1800 spectrometer equipped with a Peltier temperature controller. Steady-state fluorescence spectra were recorded on a JASCO FP-5300 fluorospectrometer equipped with a temperature controller. Obtained spectra were corrected by referencing to a tungsten sub-reference lamp (JASCO). ^1^H NMR spectra were recorded on a Bruker AVANCE DPX400 spectrometer (9.4 T) at ambient temperature in deuterated chloroform containing *ca.* 0.03% (v/v) tetramethylsilane as an internal standard (*δ* = 0.00 ppm). TEM images were obtained using an FEI Tecnai Osiris field emission transmission electron microscope with an accelerating voltage of 200 kV.

### 3.3 Synthesis

#### 3.3.1 Gold Nanoparticles A (2.5 ± 0.5 nm)

The title nanoparticles A were synthesized by one-phase method reported in the literature (Zheng et al., 2006). To a solution of chloro(triphenylphosphine)gold(I) (Braunstein et al., 2007) (250 mg, 0.505 mmol) and 1-dodecanethiol (250 mg, 1.24 mmol) in chloroform (12.5 ml), borane *tert*-butylamine complex (423 mg, 4.86 mmol) in chloroform/ethanol (12.5 ml/5 ml) was added. After 24 h of stirring at ambient temperature, the mixture was concentrated under reduced pressure, and ethanol (40 ml) was added. The precipitate was collected by centrifugation and repeatedly washed with ethanol under sonication to remove starting materials and other byproducts to obtain A as a black waxy solid (61 mg). The mean diameter and size distribution (a standard deviation) were determined to be 2.5 and 0.5 nm, respectively, by transmission electron microscopy (TEM) (Supplementary Figure S2).

#### 3.3.2 5-(4-Nitrophenyl)-10,15,20-tris(3,5-di-*tert*-butylphenyl)porphyrin (3)

Pyrrole (2.19 ml, 30.0 mmol), 4-nitrobenzaldehyde (1.14 g, 7.5 mmol), and 3,5-di-*tert*-butylbenzaldehyde (Schuster et al., 2006) (4.92 g, 22.5 mmol) were dissolved in methylene chloride (3 L) and purged with nitrogen for 15 min. To the solution, trifluoroacetic acid (4.44 ml, 60.0 mmol) was added, and the mixture was stirred for 1 h in the dark before neutralization by the addition of triethylamine (14.4 ml, 22.8 mmol). To the mixture, *p*-chloranil (5.52 g, 22.8 mmol) was added, and the solution was stirred for 3 h at ambient temperature. After the removal of the solvent under reduced pressure, the residue was purified by column chromatography (silica gel, hexane:methylene chloride = 2:1), followed by recrystallization (methylene chloride/methanol) to obtain 3 as purple crystals (1.23 g, 16%). ^1^H NMR (400 MHz, CDCl_3_, SiMe_4_, 294 K): *δ*/ppm −2.70 (2H, brs, inner NH), 1.51–1.55 (54H, m, CH_3_), 7.80 (1H, t, *J* = 1.7 Hz, benzene), 7.81 (2H, t, *J* = 1.7 Hz, benzene), 8.08 (2H, d, *J* = 1.8 Hz, benzene), 8.08 (4H, d, *J* = 1.8 Hz, benzene), 8.43 (2H, d, *J* = 8.6 Hz, benzene), 8.64 (2H, d, *J* = 8.6 Hz, benzene), 8.74 (2H, d, *J* = 4.7 Hz, pyrrole), and 8.90–8.97 (6H, m, pyrrole).

#### 3.3.3 5-(4-Aminophenyl)-10,15,20-tris(3,5-di-*tert*-butylphenyl)porphyrin (4)

To a dispersion of 3 (1.39 g, 1.40 mmol) in concentrated hydrochloric acid (50 ml), stannous chloride dihydrate (1.90 g, 8.40 mmol) was added, and the suspension was stirred for 12 h at 70°C. The mixture was neutralized by the addition of sodium carbonate and extracted with methylene chloride. The organic layer was washed with water and brine, dried over sodium sulfate, then concentrated under reduced pressure. The residue was purified by column chromatography (silica gel and dichloromethane) and then recrystallized from methylene chloride/methanol to obtain 4 as purple crystals (0.29 g, 21%). The majority of 3 was unreacted, apparently due to its low solubility. ^1^H NMR (400 MHz, CDCl_3_, SiMe_4_, 296 K): *δ*/ppm −2.69, (2H, brs, inner NH), 1.50–1.55 (54H, m, CH_3_), 4.02 (brs, 2H, NH_2_), 7.06 (2H, d, *J* = 8.2 Hz, benzene), 7.78 (1H, t, *J* = 1.7 Hz, benzene), 7.79 (2H, t, *J* = 1.7 Hz, benzene), 8.01 (2H, d, *J* = 8.2 Hz, benzene), 8.07 (2H, d, *J* = 1.7 Hz, benzene) 8.09 (4H, d, *J* = 1.7 Hz, benzene), 8.82–8.92 (6H, m, pyrrole), and 8.94 (2H, d, *J* = 4.6 Hz, pyrrole).

#### 3.3.4 6-Bromo-*N*-{4-[10,15,20-tris(3,5-di-*tert*-butylphenyl)porphyrin-5-yl]phenyl}hexanamide (5)

To a mixture of *ε*-bromocaproic acid (*ca.* 10 eq.) and *N*,*N*-dimethylformamide (1 drop) in toluene, excess thionyl chloride was added. After 1 h of stirring at ambient temperature, the mixture was concentrated under reduced pressure. The residue was redissolved in toluene and concentrated again to obtain *ε*-bromocaproyl chloride as yellow oil, which was subjected to the following reaction without purification.

An ice-cold mixture of 4 (50.0 mg, 0.0517 mmol) and dry pyridine (0.1 ml) in dry methylene chloride (5 ml), *ε*-bromocaproyl chloride (*ca.* 10 eq.) in dry methylene chloride was added dropwise. After 1 h of stirring, the mixture was concentrated under reduced pressure to obtain crude 5 as purple solid. This material was subjected to the following reaction without further purification.

#### 3.3.5 6-Acetylthio-*N*-{4-[10,15,20-tris(3,5-di-*tert*-butylphenyl)porphyrin-5-yl]phenyl}hexanamide (6)

Crude 5 was dissolved in bench *N*,*N*-dimethylformamide, and potassium thioacetate (50 mg, 0.44 mmol) was added at 0°C. After 1 h of stirring at ambient temperature, the mixture was diluted with methylene chloride (40 ml) and washed with water. The organic layer was dried over sodium sulfate and concentrated under reduced pressure. The residue was purified by column chromatography (silica gel and methylene chloride) and subsequent reprecipitation from methylene chloride/methanol to obtain 6 as purple solid. 49 mg (83% from 4). ^1^H NMR (400 MHz, CDCl_3_, SiMe_4_, 296 K): *δ*/ppm −2.71 (2H, brs, inner NH), 1.50–1.56 (54H, m, CH_3_), 1.58 (2H, quint, *J* = 7.0 Hz, CH_2_), 1.72 (2H, quint, *J* = 7.3 Hz, CH_2_), 1.91 (2H, quint, *J* = 7.5 Hz, CH_2_), 2.54 (2H, t, *J* = 7.5 Hz, NHCOCH_2_), 2.96 (2H, t, *J* = 7.3 Hz, AcSCH_2_), 7.56 (1H, brs, NHCO), 7.78 (1H, t, *J* = 1.6 Hz, benzene), 7.79 (2H, t, *J* = 1.6 Hz, benzene), 7.91 (2H, d, *J* = 8.2 Hz, benzene), 8.07 (2H, d, *J* = 1.7 Hz, benzene), 8.08 (4H, d, *J* = 1.7 Hz, benzene), 8.19 (2H, d, *J* = 8.3 Hz, benzene), 8.86 (2H, d, *J* = 4.6 Hz, pyrrole), and 8.87–8.92 (6H, m, pyrrole).

#### 3.3.6 6-Mercapto-N-{4-[10,15,20-tris(3,5-di-tert-butylphenyl)porphyrin-5-yl]phenyl}hexanamide (2)

The title compound **2** was synthesized according to the method reported in the literature (Imahori et al., 2004b). To a solution of 6 (34.0 mg, 0.0299 mmol) in degassed tetrahydrofuran (1 ml), potassium hydroxide (30 mg, 0.53 mmol) was added in degassed methanol (1 ml) and the mixture refluxed for 15 min under nitrogen. The mixture was neutralized by the addition of acetic acid (0.1 ml) and then concentrated under reduced pressure. The residue was purified by column chromatography (silica gel and methylene chloride) and subsequent reprecipitation from methylene chloride/methanol to obtain 2 as purple solid. 22 mg (66%). ^1^H NMR (400 MHz, CDCl_3_, SiMe_4_, and 296 K): *δ*/ppm −2.70 (2H, brs, inner NH), 1.41 (1H, t, *J* = 7.8 Hz, SH), 1.51–1.55 (54H, m, CH_3_), 1.60 (2H, quint, *J* = 7.1 Hz, CH_2_), 1.75 (2H, quint, *J* = 7.3 Hz, CH_2_), 1.88 (2H, quint, *J* = 7.5 Hz, CH_2_), 2.52 (2H, t, *J* = 7.4 Hz, NHCOCH_2_), 2.62 (2H, q, *J* = 7.2 Hz, HSCH_2_), 7.43 (1H, brs, NHCO), 7.78 (1H, t, *J* = 1.8 Hz, benzene), 7.79 (2H, t, *J* = 1.8 Hz, benzene), 7.90 (2H, d, *J* = 8.3 Hz, benzene), 8.07 (2H, d, *J* = 1.8 Hz, benzene), 8.08 (4H, d, *J* = 1.9 Hz, benzene), 8.15–8.19 (2H, d, 8.4 Hz, benzene), 8.85 (2H, d, *J* = 4.7 Hz, pyrrole), and 8.87–8.93 (6H, m, pyrrole).

#### 3.3.7 Porphyrin–AuNP conjugates (1a@A and 2@A)

The solution of A (1 mg/ml = 9.6 μM, assuming the chemical formula of Au_400_(C_12_H_25_S)_126_ = 1.04 × 10^5^ Da (Terrill et al., 1995)) and porphyrin–alkanethiol (i.e*.*, 1b or 2, 15–50 mM) in toluene (1 ml), was allowed to equilibrate for 24 h at ambient temperature. The mixture was concentrated under reduced pressure at ambient temperature using a centrifugal evaporator (EYELA CVE-2000 equipped with a common oil rotary vacuum pump). The residue was purified twice by size exclusion chromatography (BioBeads S-X1, toluene) to remove unreacted porphyrin–alkanethiol.

### 3.4 UV-Vis Extinction Spectra

Purified porphyrin–AuNP conjugates were dissolved in toluene to achieve an absorbance at 515 nm of 0.20 ± 0.01 [−], and extinction spectra were recorded. The same solution was subjected to fluorescence measurement (*vide infra*).

The molar extinction coefficients (*ε*) of AuNPs are related to their size and surface dielectric constant (Huang and El-Sayed, 2010). In the thiol/thiolate exchange reaction, no significant size change occurs, or even if it does occur, its effect is negligible (Hostetler et al., 1999). The surface dielectric constant mainly reflects the structure of the adsorbates, but since both the original 1-dodecanethiolate and the porphyrin–alkanethiolate are similar, the surface dielectric constants are unlikely to change. Assuming that the porphyrin–alkanethiolate adsorbates are also not affected by the AuNPs, the extinction spectrum of the porphyrin–AuNP conjugates can be considered as the sum of the extinction spectra of the 1-dodecanethiolate–protected AuNPs and the porphyrin–alkanethiol.

Based on this assumption, the extinction spectra of porphyrin–AuNP conjugates were deconvoluted using the following equation (Shinohara and Shinmori, 2016; Shinohara et al., 2020):

$$A\left(\lambda \right)=a\times \varepsilon_{\text{Por}}\left(\lambda \right)+b\times \varepsilon_{\text{AuNP}}\left(\lambda \right)+R\left(\lambda \right).$$ (1)

Here, *A*(*λ*) is the actual extinction spectrum of porphyrin–AuNP conjugates. *ε*_Por_(*λ*) and *ε*_AuNP_(*λ*) are the separately measured molar absorption coefficients of porphyrin–alkanethiol (1a–c or 2) and 1-dodecanethiolate-protected AuNPs (A–C), respectively. The proportional coefficients *a* and *b* were determined by the non-linear least-squares method (300 ≦ *λ* ≦ 800 nm), and the residue *R*(*λ*) was obtained. Finally, to obtain the number of porphyrin–alkanethiolate adsorbates per particle (*N*), as the quotient of the coefficients *a* and *b*, the following equation was used:

N=a/b. (2)

To qualify the aggregation of porphyrin adsorbates on AuNPs, *ε*_Por_(*λ*) was further deconvoluted into two Gaussian functions, and the relative intensity of shoulder to the Soret band ( $\varepsilon_{\text{shoulder}}/\varepsilon_{\text{Soret}}$ ) was obtained (Supplementary Tables S3, S4, S13–S18).

### 3.5 Fluorescence Spectra

Five milliliters of the solution of porphyrin–AuNP conjugates, used for the extinction spectra measurement (absorbance at 515 nm–0.2), was volumetrically diluted by toluene to make 50 ml. The absorbance of the solution was reduced to below 0.02 (>95% transmittance) in the measurement range, to ensure the elimination of reabsorption of fluorescence by the sample itself. *Φ*_F_ was determined by the following equation (Zhang et al., 2014):

$$\Phi_{\text{F}}=\Phi_{\text{F},\text{ref}}\times \frac{A_{\text{ref}}}{A}\times \frac{S}{S_{\text{ref}}},$$ (3)

where *Φ*_F,ref_ shows *Φ*_F_ of *meso*-tetraphenylporphyrin (10^–8^ M, *Φ*_F,ref_ = 0.11 (Seybold and Gouterman, 1969)) as an external standard, and *A* and *S* show absorbance of porphyrin adsorbates at an excitation wavelength (515 nm) and the area of fluorescence spectra (600–800 nm), respectively.

## 4 Conclusion

In this work, a series of porphyrin–alkanethiols that have different linker chain lengths and substituents were loaded on the AuNPs by the post-synthetic thiol/thiolate exchange reaction. The conditions of the reaction were optimized to obtain low loading levels of total thiolate sites on the AuNPs. The conjugates retain the unique photoabsorption features of porphyrin (the Soret band and Q band) after loading. Slight red-shift, shoulder growth, and broadening were observed in the Soret band but not in the Q bands. Interestingly, porphyrin adsorbates form *H*-aggregates soon after the onset of the thiol/thiolate exchange reaction. Fluorescence in the adsorbates was determined to be strongly quenched, due to intraparticle energy transfer from the excited adsorbates to the AuNPs. Contrary to our hypothesis, the aggregation contributes little to the fluorescence quenching. Fluorescence quantum yields are affected by linker chain length but neither by the loading level nor nanoparticle diameter, unlike our previous report on the singlet oxygen quantum yields. Thus, this work has advanced our understanding of the photophysical properties of dye adsorbates on AuNPs.

## Data Availability

The raw data supporting the conclusion of this article will be made available by the authors, without undue reservation.
