# Supplementary material for: Porphyrin Photoabsorption and Fluorescence Variation with Adsorptive Loading on Gold Nanoparticles
Source: Front Chem. 2021 Nov 23;9:777041. doi: 10.3389/fchem.2021.777041 (PMC8650619; doi:10.3389/fchem.2021.777041)
Supplement: Supplementary file 1 [file DataSheet1.docx]

Supplementary Material for

Porphyrin Photoabsorption and Fluorescence Variation with Adsorptive Loading on Gold Nanoparticles

Akira Shinohara^1,2,3^, Guang Shao^1^, Takashi Nakanishi^3^*, Hideyuki Shinmori^2^*

^1^School of Chemistry, Sun Yat-sen University, Guangzhou 510006, China

^2^Department of Biotechnology, Faculty of Life and Environmental Science, Graduate Faculty of Interdisciplinary Research, University of Yamanashi, Kofu 400-8510, Japan

^3^International Center for Materials Nanoarchitectonics (WPI-MANA), National Institute for Materials Science (NIMS), Tsukuba 305-0044, Japan


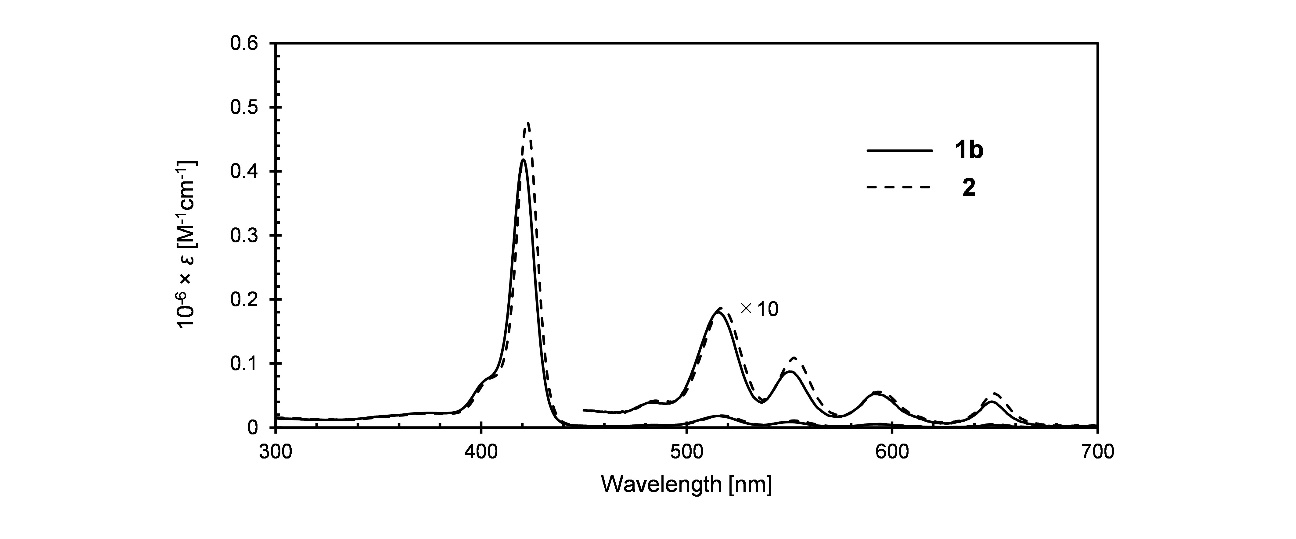


Figure S1. UV–vis absorption spectra of 1b and 2 in toluene.


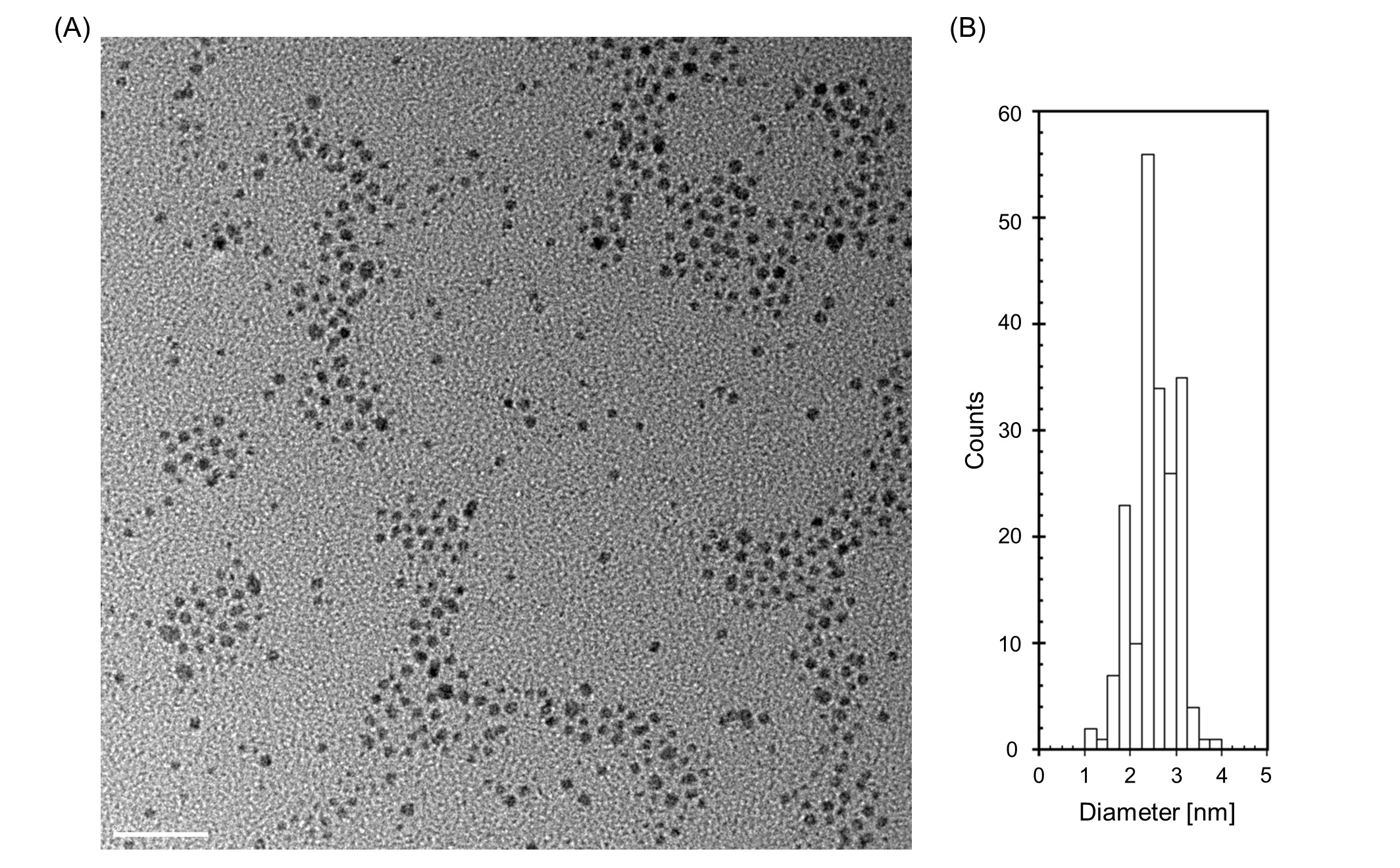


**Figure S2.** (A) TEM image (B) and size distribution (200 particle counts) of 1-dodecanethiolate-protedted gold nanoparticles **A** (2.5 ± 0.5 nm). Scale bar = 20 nm.

**Synthesis of 1a–c@B and 1a–c@C** (Shinohara and Shinmori, 2016)

To the toluene solution of **B** (Au_400_(C_12_H_25_S)_126_ = 1.04 × 10^5^ Da, 10 mg/mL, 1 mL, 96 nmol) was added porphyrin–alkanethiol (5 mg, **1a**: 7.0 μmol, **1b**: 6.6 μmol, **1c**: 6.0 μmol) and the mixture was sonicated to make homogeneous. After specific reaction time (Entry 1: *t* = 5 min, Entry 2: 10 min, Entry 3: 15 min, Entry 4: 30 min, Entry 5: 60 min), the aliquots (50 μL) were added to the excess acetone (10 mL) and centrifuged (10,000 ×*g*). The precipitate was washed with acetone and purified twice by size exclusion chromatography (BioBeads S-X1, toluene) to remove unreacted porphyrin–alkanethiol.

**1a–c@C** were synthesized using similar method, except for the **C** (assumed formula Au_4500_(C_12_H_25_S)_632_ = 1.01× 10^6^ kDa, 10 mg/mL, 1 mL, 9.9 nmol) was used as precursor and extention of reaction time (Entry 1: *t* = 10 min, Entry 2: 20 min, Entry 3: 30 min, Entry 4: 60 min, Entry 5: 90 min).


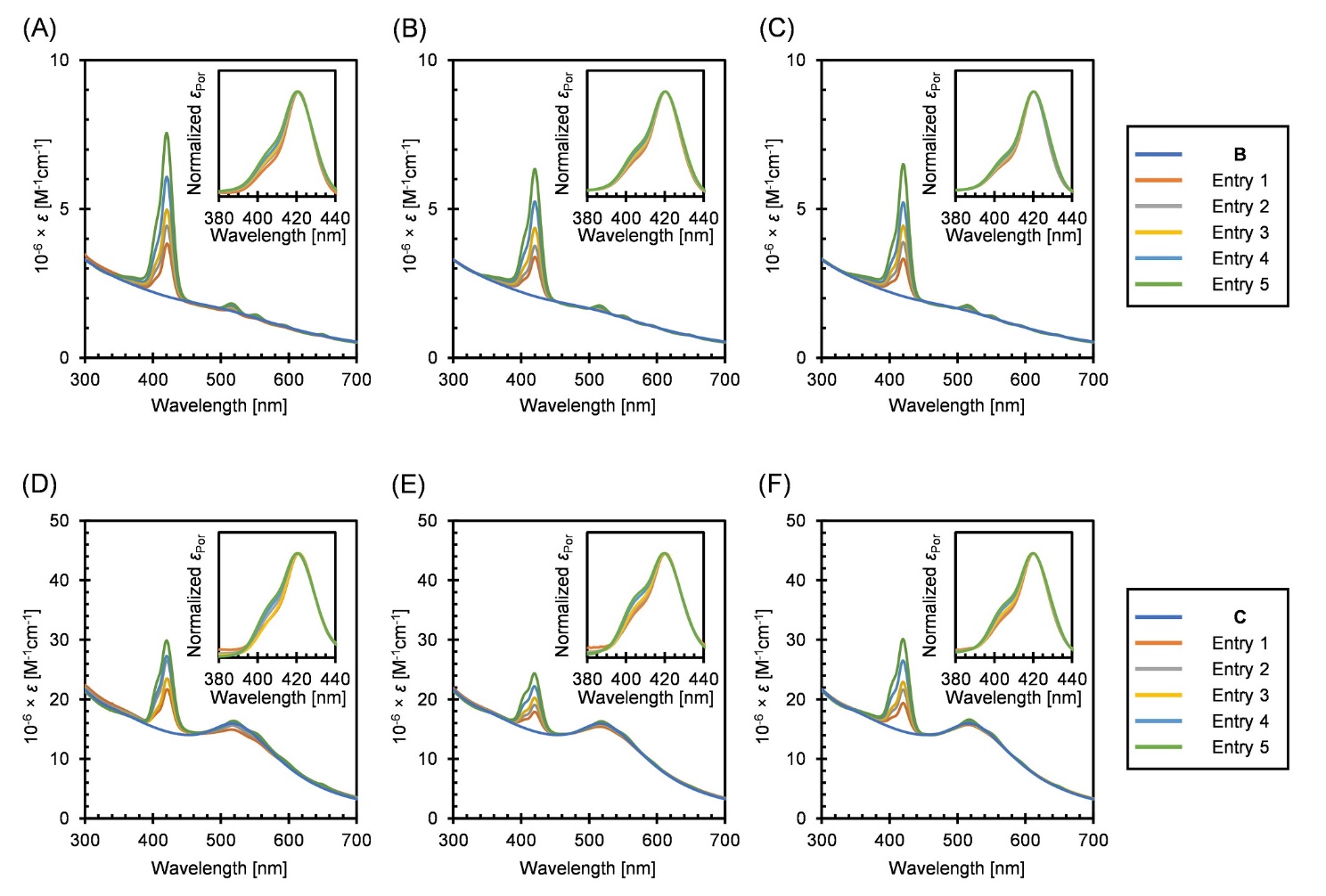


**Figure S3.** UV–vis extinction spectra (in toluene) of (A) **1a@B**, (B) **1b@B**, (C) **1c@B**, (D) **1a@C**, (E) **1b@C**, and (F) **1c@C** prepared by post-synthetic modification of nanoparticle **B**. Insets represent normalized absorption spectra of porphyrin adsorbates.

**
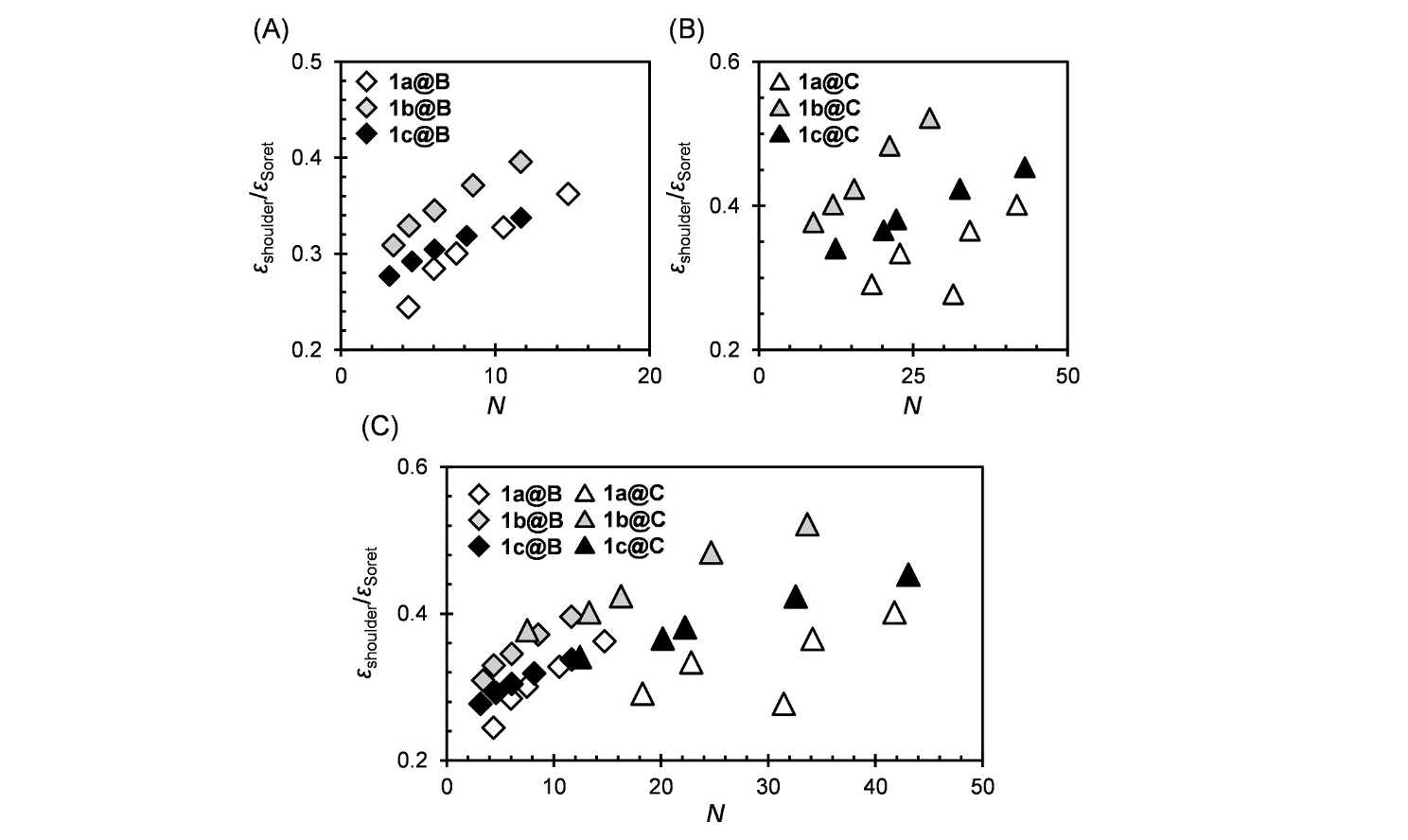
**

**Figure S4.** Relative shoulder intensity ($\varepsilon_{\mathrm{shoulder}}/\varepsilon_{\mathrm{Soret}}$) of porphyrin adsorbates on (A) **B** and (B) **C** with different numbers of porphyrin–alkanethiolate adsorbates per particle (*N*). (C) Overlayed plot of (A) and (B).

**
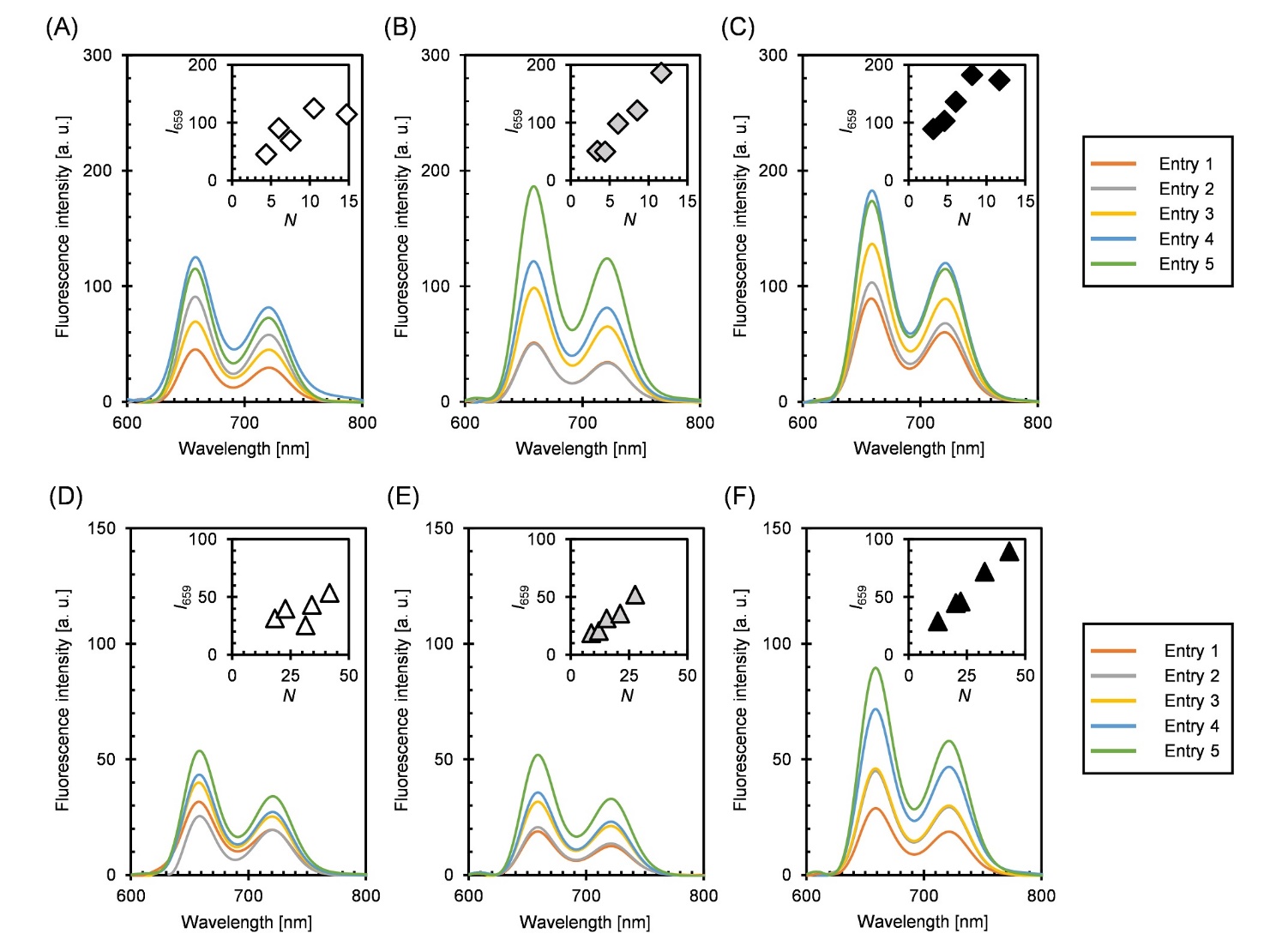
**

**Figure S5.** Steady-state fluorescence spectra of (A) **1a@B**, (B) **1b@B**, (C) **1c@B**, (D) **1a@C**, (E) **1b@C**, and (F) **1c@C** in toluene (saturated with air at *p*O_2_ = *ca.* 0.21 atm, *λ*_ex_ = 515 nm). Insets show fluorescence intensity at 659 nm (*I*_659_, Q_(0-0)_ band) versus *N*.

**
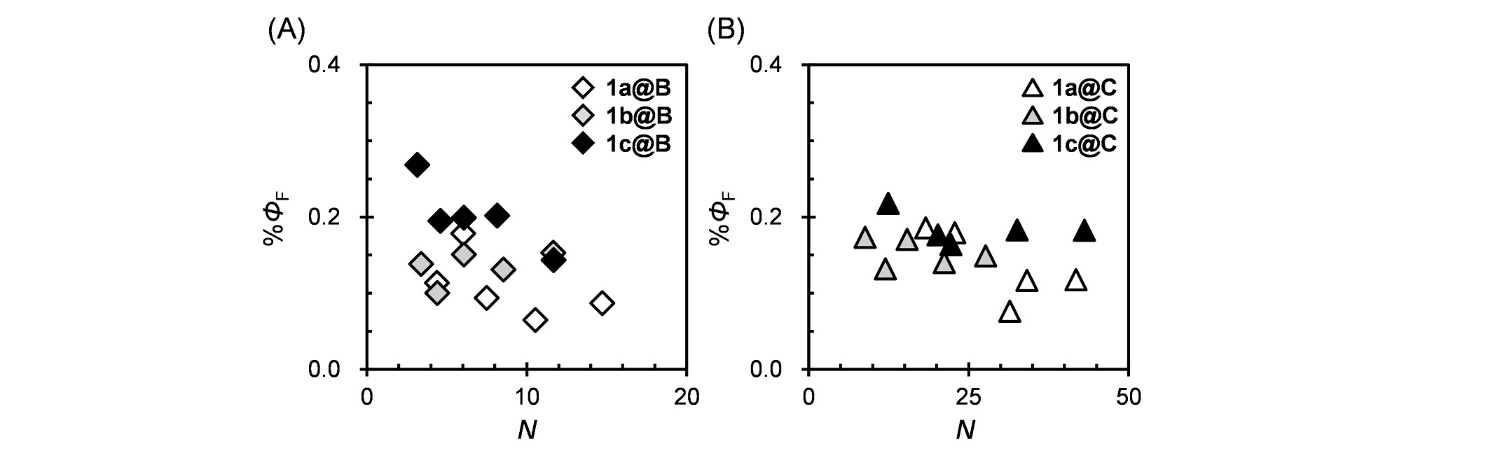
**

**Figure S6.** Fluorescence quantum yields (*Φ*_F_) of (A) **1a**–**c@B** and (B) **1a**–**c@C** with different *N*.


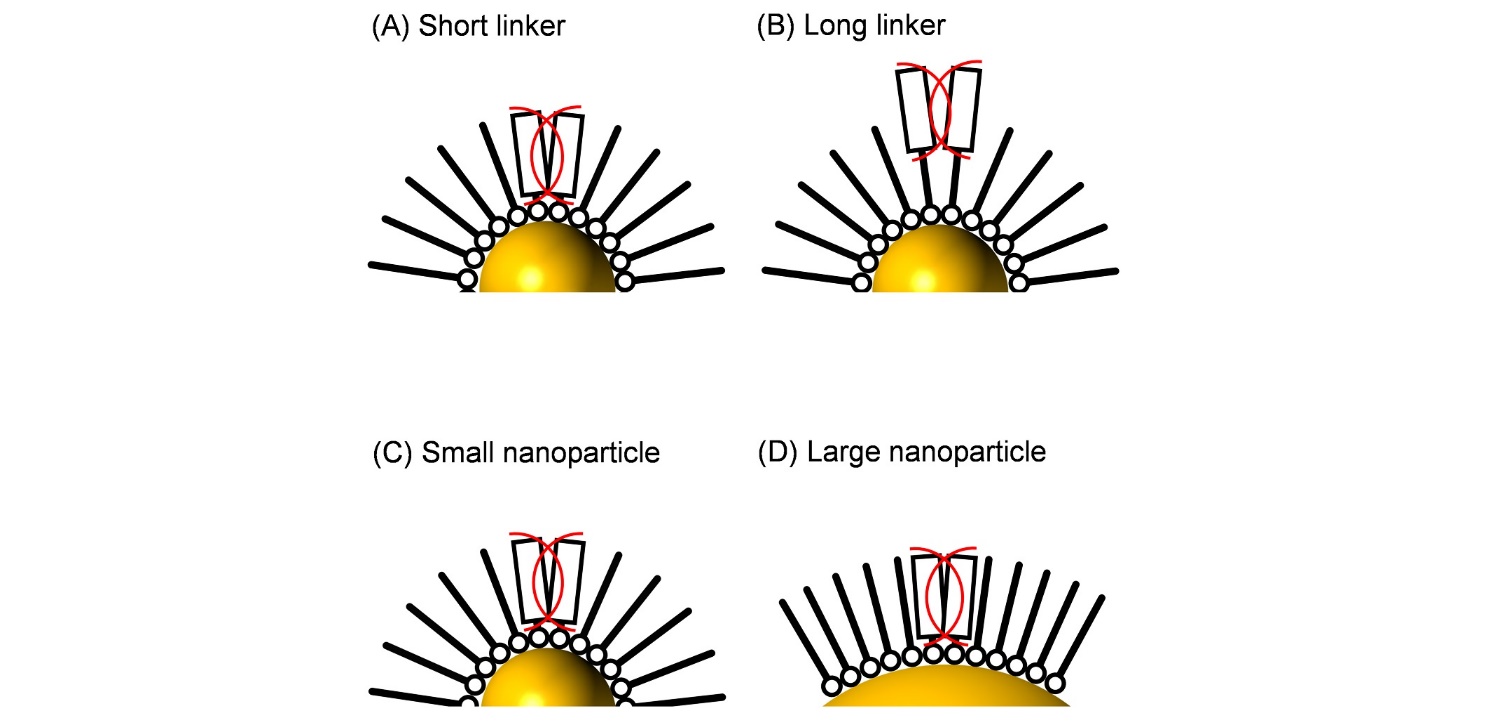


**Figure S7.** Schematic illustration of steric hindrance between adjacent porphyrins with (A, B) different linker chain lengths and (C, D) different nanoparticle diameters.


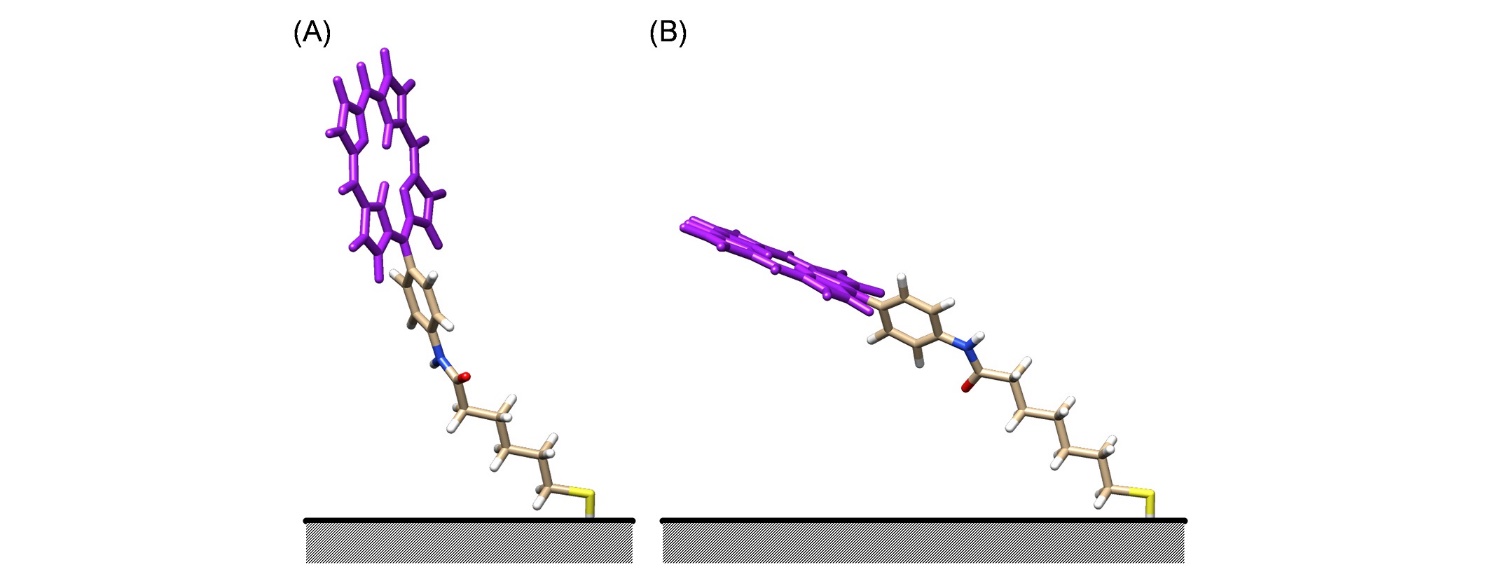


**Figure S8.** Possible porphyrin orientation on two-dimensional Au substrate for chains with (A) an odd and (B) even number of methylene linkers (Imahori et al., 2000).

**Table S1.** Absorption and emission maxima of **1b@A** in toluene.

| *c*_0_  [mM] | Absorption maxima  [nm] | | | | | Emission maxima  [nm] | |
| --- | --- | --- | --- | --- | --- | --- | --- |
|  | B_(0,0)_ | Q_y(1,0)_ | Q_y(0,0)_ | Q_x(1,0)_ | Q_x(0,0)_ | Q_(0,0)_ | Q_(1,0)_ |
| (**1b**) | 421 | 515 | 550 | 591 | 648 | 654 | 720 |
| 15 | 421 | 519 | 552 | 593 | 648 | 658 | 722 |
| 20 | 421 | 519 | 552 | 593 | 648 | 658 | 722 |
| 25 | 421 | 519 | 552 | 593 | 648 | 658 | 722 |
| 30 | 420 | 517 | 552 | 593 | 648 | 658 | 722 |
| 35 | 420 | 518 | 554 | 593 | 648 | 658 | 721 |
| 40 | 420 | 518 | 553 | 592 | 649 | 658 | 721 |
| 45 | 420 | 517 | 552 | 592 | 649 | 658 | 721 |
| 50 | 420 | 518 | 554 | 594 | 650 | 658 | 721 |

**Table S2.** Absorption and emission maxima of **2@A** in toluene.

| *c*_0_  [mM] | Absorption maxima  [nm] | | | | | Emission maxima  [nm] | |
| --- | --- | --- | --- | --- | --- | --- | --- |
|  | B_(0,0)_ | Q_y(1,0)_ | Q_y(0,0)_ | Q_x(1,0)_ | Q_x(0,0)_ | Q_(0,0)_ | Q_(1,0)_ |
| (**2**) | 423 | 517 | 552 | 593 | 650 | 655 | 721 |
| 15 | 422 | 518 | 555 | 593 | 649 | 658 | 722 |
| 20 | 422 | 518 | 555 | 593 | 649 | 658 | 722 |
| 25 | 422 | 518 | 555 | 593 | 649 | 658 | 722 |
| 30 | 422 | 518 | 554 | 594 | 651 | 658 | 722 |
| 35 | 421 | 518 | 554 | 594 | 651 | 658 | 722 |
| 40 | 421 | 517 | 554 | 594 | 650 | 658 | 722 |
| 45 | 421 | 518 | 554 | 594 | 651 | 658 | 722 |
| 50 | 421 | 518 | 554 | 594 | 651 | 658 | 722 |

**Table S3.** Fitting parameters for Soret band of **1b@A**

| Parameter | *c*_0_ [mM] | | | | | | | | |
| --- | --- | --- | --- | --- | --- | --- | --- | --- | --- |
|  | (**1b**) | 15 | 20 | 25 | 30 | 35 | 40 | 45 | 50 |
| *A*_0_ | 0.031 | 0.018 | 0.022 | 0.026 | 0.026 | 0.026 | 0.030 | 0.028 | 0.030 |
| *A*_1_ | 0.849 | 0.970 | 0.965 | 0.960 | 0.957 | 0.955 | 0.950 | 0.948 | 0.944 |
| *μ*_1_ | 419.0 | 421.5 | 421.3 | 421.2 | 421.1 | 420.6 | 420.6 | 420.7 | 420.6 |
| *σ* _1_ | 5.19 | 7.24 | 7.28 | 7.35 | 7.39 | 7.64 | 7.71 | 7.71 | 7.84 |
| *A*_2_ | 0.123 | 0.197 | 0.219 | 0.240 | 0.258 | 0.291 | 0.302 | 0.315 | 0.332 |
| *μ*_2_ | 403.0 | 403.4 | 403.5 | 403.5 | 403.6 | 403.3 | 403.3 | 403.5 | 403.5 |
| *σ*_2_ | 7.38 | 5.89 | 6.13 | 6.34 | 6.56 | 6.37 | 6.51 | 6.75 | 6.88 |
| FWHM | 12.2 | 17.0 | 17.1 | 17.3 | 17.4 | 18.0 | 18.2 | 18.2 | 18.5 |
| *ε*_shoulder_/*ε*_Soret_ | 0.145 | 0.211 | 0.236 | 0.260 | 0.279 | 0.332 | 0.328 | 0.342 | 0.362 |
| $\varepsilon_{\mathrm{Por}}(\lambda) = A_{0}+\Sigma\{A_{i}\times exp(-{(\lambda-\mu_{i})}^{2}/2\sigma_{i}^{2})\}$  $\varepsilon_{\mathrm{shoulder}}/\varepsilon_{\mathrm{Soret}}=A_{2}/A_{1}$  $\mathrm{FWHM}=2\sqrt{2ln2} \sigma_{1}$ | | | | | | | | | |

**Table S4.** Fitting parameters for Soret band of **2@A**

| Parameter | *c*_0_ [mM] | | | | | | | | |
| --- | --- | --- | --- | --- | --- | --- | --- | --- | --- |
|  | (**2**) | 15 | 20 | 25 | 30 | 35 | 40 | 45 | 50 |
| *A*_0_ | 0.035 | 0.024 | 0.026 | 0.028 | 0.028 | 0.028 | 0.029 | 0.029 | 0.029 |
| *A*_1_ | 1.08 | 0.962 | 0.962 | 0.963 | 0.963 | 0.962 | 0.963 | 0.963 | 0.963 |
| *μ*_1_ | 421.5 | 422.4 | 422.2 | 422.0 | 421.9 | 421.8 | 421.7 | 421.6 | 421.5 |
| *σ* _1_ | 4.79 | 6.01 | 6.07 | 6.16 | 6.22 | 6.27 | 6.32 | 6.37 | 6.40 |
| *A*_2_ | 0.149 | 0.141 | 0.145 | 0.150 | 0.154 | 0.158 | 0.161 | 0.164 | 0.166 |
| *μ*_2_ | 406.2 | 404.4 | 404.2 | 404.1 | 404.0 | 403.9 | 403.8 | 403.8 | 403.8 |
| *σ*_2_ | 7.71 | 5.45 | 5.46 | 5.44 | 5.46 | 5.44 | 5.45 | 5.45 | 5.48 |
| FWHM | 11.3 | 14.2 | 14.3 | 14.5 | 14.6 | 14.8 | 14.9 | 15.0 | 15.1 |
| *ε*_shoulder_/*ε*_Soret_ | 0.138 | 0.157 | 0.162 | 0.168 | 0.172 | 0.176 | 0.182 | 0.184 | 0.202 |
| $\varepsilon_{\mathrm{Por}}(\lambda) = A_{0}+\Sigma\{A_{i}\times exp(-{(\lambda-\mu_{i})}^{2}/2\sigma_{i}^{2})\}$  $\varepsilon_{\mathrm{shoulder}}/\varepsilon_{\mathrm{Soret}}=A_{2}/A_{1}$  $\mathrm{FWHM}=2\sqrt{2ln2} \sigma_{1}$ | | | | | | | | | |

**Table S5.** Rejection test (95.4% confidence interval) for the *Φ*_F_ of **1b@A** and **2@A**

| *c*_0_ [mM] | *Φ*_F_ on AuNP [%] | |
| --- | --- | --- |
|  | **1b@A** | **2@A** |
| 15 | 0.102 | 0.180 |
| 20 | 0.143 | 0.168 |
| 25 | 0.114 | 0.247 |
| 30 | 0.095 | 0.150 |
| 35 | 0.109 | 0.145 |
| 40 | 0.108 | 0.124 |
| 45 | 0.077 | 0.137 |
| 50 | 0.077 | 0.160 |
| average | 0.103 | 0.152 |
| 2SD | 0.040 | 0.035 |
| ■ Rejected  ■ Not rejected | | |

**Table S6.** *p*-Values for Welch t-statistics between the average *Φ*_F_

|  | **1b@A** | **2@A** | **1a@B** | **1b@B** | **1c@B** | **1a@C** | **1b@C** | **1c@C** |
| --- | --- | --- | --- | --- | --- | --- | --- | --- |
| **1b@A** |  |  |  |  |  |  |  |  |
| **2@A** | 0.002 |  |  |  |  |  |  |  |
| **1a@B** | 0.857 | 0.044 |  |  |  |  |  |  |
| **1b@B** | 0.031 | 0.100 | 0.255 |  |  |  |  |  |
| **1c@B** | 0.005 | 0.159 | 0.009 | 0.024 |  |  |  |  |
| **1a@C** | 0.210 | 0.279 | 0.356 | 0.980 | 0.050 |  |  |  |
| **1b@C** | 0.001 | 0.497 | 0.078 | 0.181 | 0.071 | 0.460 |  |  |
| **1c@C** | 0.000 | 0.225 | 0.012 | 0.005 | 0.474 | 0.076 | 0.031 |  |
| ■ Means are statistically equivalent ($p>0.05$)  ■ Means are not statistically equivalent ($p\leq0.05$) | | | | | | | | |

**Table S7.** Absorption and emission maxima of **1a@B** in toluene.

| Entry | Absorption maxima  [nm] | | | | | Emission maxima  [nm] | |
| --- | --- | --- | --- | --- | --- | --- | --- |
|  | B_(0,0)_ | Q_y(1,0)_ | Q_y(0,0)_ | Q_x(1,0)_ | Q_x(0,0)_ | Q_(0,0)_ | Q_(1,0)_ |
| (**1a**) | 421 | 515 | 550 | 591 | 648 | 654 | 720 |
| 1 | 421 | 517 | 553 | 595 | 650 | 657 | 719 |
| 2 | 421 | 517 | 553 | 594 | 651 | 657 | 719 |
| 3 | 421 | 517 | 553 | 595 | 651 | 657 | 719 |
| 4 | 421 | 517 | 553 | 594 | 651 | 656 | 719 |
| 5 | 421 | 517 | 553 | 594 | 651 | 656 | 718 |

**Table S8.** Absorption and emission maxima of **1b@B** in toluene.

| Entry | Absorption maxima  [nm] | | | | | Emission maxima  [nm] | |
| --- | --- | --- | --- | --- | --- | --- | --- |
|  | B_(0,0)_ | Q_y(1,0)_ | Q_y(0,0)_ | Q_x(1,0)_ | Q_x(0,0)_ | Q_(0,0)_ | Q_(1,0)_ |
| (**1b**) | 421 | 515 | 550 | 591 | 648 | 654 | 720 |
| 1 | 421 | 516 | 553 | 594 | 650 | 657 | 719 |
| 2 | 421 | 517 | 553 | 594 | 651 | 657 | 719 |
| 3 | 421 | 517 | 554 | 594 | 649 | 657 | 719 |
| 4 | 420 | 516 | 553 | 593 | 651 | 656 | 719 |
| 5 | 420 | 517 | 553 | 594 | 650 | 657 | 719 |

**Table S9.** Absorption and emission maxima of **1c@B** in toluene.

| Entry | Absorption maxima  [nm] | | | | | Emission maxima  [nm] | |
| --- | --- | --- | --- | --- | --- | --- | --- |
|  | B_(0,0)_ | Q_y(1,0)_ | Q_y(0,0)_ | Q_x(1,0)_ | Q_x(0,0)_ | Q_(0,0)_ | Q_(1,0)_ |
| (**1c**) | 421 | 515 | 550 | 591 | 648 | 654 | 720 |
| 1 | 420 | 516 | 552 | 594 | 650 | 657 | 719 |
| 2 | 420 | 516 | 552 | 594 | 651 | 657 | 719 |
| 3 | 420 | 516 | 552 | 594 | 649 | 657 | 719 |
| 4 | 420 | 516 | 553 | 593 | 649 | 657 | 719 |
| 5 | 420 | 516 | 553 | 594 | 649 | 657 | 719 |

**Table S10.** Absorption and emission maxima of **1a@C** in toluene.

| Entry | Absorption maxima  [nm] | | | | | Emission maxima  [nm] | |
| --- | --- | --- | --- | --- | --- | --- | --- |
|  | B_(0,0)_ | Q_y(1,0)_ | Q_y(0,0)_ | Q_x(1,0)_ | Q_x(0,0)_ | Q_(0,0)_ | Q_(1,0)_ |
| (**1a**) | 421 | 515 | 550 | 591 | 648 | 654 | 720 |
| 1 | 421 | 516 | 554 | 597 | 652 | 657 | 719 |
| 2 | 421 | 517 | 558 | 597 | 652 | 657 | 719 |
| 3 | 421 | 518 | 558 | 596 | 651 | 656 | 719 |
| 4 | 421 | 518 | 557 | 596 | 651 | 657 | 718 |
| 5 | 421 | 520 | 558 | 596 | 651 | 657 | 719 |

**Table S11.** Absorption and emission maxima of **1b@C** in toluene.

| Entry | Absorption maxima  [nm] | | | | | Emission maxima  [nm] | |
| --- | --- | --- | --- | --- | --- | --- | --- |
|  | B_(0,0)_ | Q_y(1,0)_ | Q_y(0,0)_ | Q_x(1,0)_ | Q_x(0,0)_ | Q_(0,0)_ | Q_(1,0)_ |
| (**1b**) | 421 | 515 | 550 | 591 | 648 | 654 | 720 |
| 1 | 421 | 518 | 559 | 599 | 653 | 657 | 719 |
| 2 | 420 | 518 | 558 | 599 | 652 | 657 | 719 |
| 3 | 420 | 518 | 558 | 599 | 651 | 657 | 719 |
| 4 | 420 | 517 | 557 | 598 | 651 | 657 | 719 |
| 5 | 420 | 518 | 557 | 598 | 651 | 657 | 719 |

**Table S12.** Absorption and emission maxima of **1c@C** in toluene.

| Entry | Absorption maxima  [nm] | | | | | Emission maxima  [nm] | |
| --- | --- | --- | --- | --- | --- | --- | --- |
|  | B_(0,0)_ | Q_y(1,0)_ | Q_y(0,0)_ | Q_x(1,0)_ | Q_x(0,0)_ | Q_(0,0)_ | Q_(1,0)_ |
| (**1c**) | 421 | 515 | 550 | 591 | 648 | 654 | 720 |
| 1 | 420 | 516 | 558 | 595 | 651 | 657 | 719 |
| 2 | 420 | 516 | 557 | 595 | 651 | 657 | 719 |
| 3 | 420 | 516 | 556 | 595 | 652 | 657 | 719 |
| 4 | 420 | 516 | 555 | 594 | 651 | 657 | 719 |
| 5 | 420 | 517 | 555 | 594 | 651 | 657 | 719 |

**Table S13.** Fitting parameters for Soret band of **1a@B**

| Parameter | Entry | | | | |
| --- | --- | --- | --- | --- | --- |
|  | 1 | 2 | 3 | 4 | 5 |
| *A*_1_ | 0.403 | 0.632 | 0.802 | 1.23 | 1.91 |
| *μ*_1_ | 405.3 | 407.3 | 406.5 | 407.0 | 407.9 |
| *σ* _1_ | 12.4 | 15.8 | 15.0 | 15.3 | 15.9 |
| *A*_2_ | 1.68 | 2.05 | 2.56 | 3.44 | 4.46 |
| *μ*_2_ | 421.9 | 422.2 | 422.1 | 422.1 | 422.2 |
| *σ*_2_ | 9.66 | 10.0 | 10.2 | 10.4 | 10.7 |
| *ε*_shoulder_/*ε*_Soret_ | 0.245 | 0.284 | 0.301 | 0.328 | 0.362 |
| $\varepsilon_{\mathrm{Por}}(\lambda) = \Sigma\{A_{i}\times exp(-{(\lambda-\mu_{i})}^{2}/2\sigma_{i}^{2})\}$  $\varepsilon_{\mathrm{shoulder}}/\varepsilon_{\mathrm{Soret}}=A_{2}/A_{1}$ | | | | | |

**Table S14.** Fitting parameters for Soret band of **1b@B**

| Parameter | Entry | | | | |
| --- | --- | --- | --- | --- | --- |
|  | 1 | 2 | 3 | 4 | 5 |
| *A*_1_ | 0.367 | 0.543 | 0.762 | 1.07 | 1.53 |
| *μ*_1_ | 405.6 | 408.5 | 408.3 | 406.2 | 406.4 |
| *σ* _1_ | 15.2 | 17.6 | 17.3 | 15.2 | 15.3 |
| *A*_2_ | 1.16 | 1.34 | 1.83 | 2.75 | 3.65 |
| *μ*_2_ | 421.6 | 422.0 | 422.1 | 421.9 | 422.0 |
| *σ*_2_ | 9.93 | 9.90 | 10.2 | 10.5 | 10.7 |
| *ε*_shoulder_/*ε*_Soret_ | 0.309 | 0.329 | 0.345 | 0.371 | 0.396 |
| $\varepsilon_{\mathrm{Por}}(\lambda) = \Sigma\{A_{i}\times exp(-{(\lambda-\mu_{i})}^{2}/2\sigma_{i}^{2})\}$  $\varepsilon_{\mathrm{shoulder}}/\varepsilon_{\mathrm{Soret}}=A_{2}/A_{1}$ | | | | | |

**Table S15.** Fitting parameters for Soret band of **1c@B**

| Parameter | Entry | | | | |
| --- | --- | --- | --- | --- | --- |
|  | 1 | 2 | 3 | 4 | 5 |
| *A*_1_ | 1.51 | 0.311 | 0.464 | 0.666 | 0.883 |
| *μ*_1_ | 404.9 | 405.5 | 404.8 | 407.7 | 405.4 |
| *σ* _1_ | 14.1 | 16.2 | 15.0 | 17.9 | 15.3 |
| *A*_2_ | 4.62 | 1.10 | 1.63 | 1.93 | 2.79 |
| *μ*_2_ | 421.5 | 421.4 | 421.3 | 421.6 | 421.4 |
| *σ*_2_ | 10.0 | 9.39 | 9.65 | 9.74 | 9.95 |
| *ε*_shoulder_/*ε*_Soret_ | 0.3427778 | 0.2770375 | 0.2920042 | 0.3044004 | 0.3186945 |
| $\varepsilon_{\mathrm{Por}}(\lambda) = \Sigma\{A_{i}\times exp(-{(\lambda-\mu_{i})}^{2}/2\sigma_{i}^{2})\}$  $\varepsilon_{\mathrm{shoulder}}/\varepsilon_{\mathrm{Soret}}=A_{2}/A_{1}$ | | | | | |

**Table S16.** Fitting parameters for Soret band of **1a@C**

| Parameter | Entry | | | | |
| --- | --- | --- | --- | --- | --- |
|  | 1 | 2 | 3 | 4 | 5 |
| *A*_1_ | 2.76 | 2.67 | 1.67 | 2.93 | 3.81 |
| *μ*_1_ | 416.8 | 402.2 | 402.3 | 402.4 | 402.3 |
| *σ* _1_ | 23.3 | 8.87 | 7.63 | 7.74 | 7.85 |
| *A*_2_ | 4.44 | 11.5 | 8.60 | 12.3 | 14.8 |
| *μ*_2_ | 422.1 | 421.2 | 421.5 | 420.8 | 420.5 |
| *σ*_2_ | 8.84 | 12.4 | 12.3 | 12.3 | 13.3 |
| *ε*_shoulder_/*ε*_Soret_ | 0.291 | 0.334 | 0.277 | 0.366 | 0.402 |
| $\varepsilon_{\mathrm{Por}}(\lambda) = \Sigma\{A_{i}\times exp(-{(\lambda-\mu_{i})}^{2}/2\sigma_{i}^{2})\}$  $\varepsilon_{\mathrm{shoulder}}/\varepsilon_{\mathrm{Soret}}=A_{2}/A_{1}$ | | | | | |

**Table S17.** Fitting parameters for Soret band of **1b@C**

| Parameter | Entry | | | | |
| --- | --- | --- | --- | --- | --- |
|  | 1 | 2 | 3 | 4 | 5 |
| *A*_1_ | 1.58 | 1.23 | 1.63 | 2.50 | 3.48 |
| *μ*_1_ | 415.1 | 401.9 | 401.9 | 401.9 | 401.9 |
| *σ* _1_ | 22.4 | 10.2 | 9.85 | 9.58 | 9.35 |
| *A*_2_ | 1.79 | 4.22 | 5.38 | 7.26 | 9.38 |
| *μ*_2_ | 421.3 | 420.6 | 420.5 | 420.2 | 420.1 |
| *σ*_2_ | 8.42 | 12.3 | 12.4 | 12.7 | 12.9 |
| *ε*_shoulder_/*ε*_Soret_ | 0.377 | 0.402 | 0.424 | 0.483 | 0.522 |
| $\varepsilon_{\mathrm{Por}}(\lambda) = \Sigma\{A_{i}\times exp(-{(\lambda-\mu_{i})}^{2}/2\sigma_{i}^{2})\}$  $\varepsilon_{\mathrm{shoulder}}/\varepsilon_{\mathrm{Soret}}=A_{2}/A_{1}$ | | | | | |

**Table S18.** Fitting parameters for Soret band of **1c@C**

| Parameter | Entry | | | | |
| --- | --- | --- | --- | --- | --- |
|  | 1 | 2 | 3 | 4 | 5 |
| *A*_1_ | 2.00 | 3.360 | 4.180 | 4.23 | 5.59 |
| *μ*_1_ | 414.0 | 414.2 | 414.3 | 405.0 | 403.3 |
| *σ* _1_ | 21.4 | 19.9 | 19.8 | 13.9 | 12.0 |
| *A*_2_ | 2.91 | 3.96 | 4.51 | 10.4 | 14.5 |
| *μ*_2_ | 421.2 | 421.4 | 421.5 | 421.5 | 421.1 |
| *σ*_2_ | 8.41 | 8.36 | 8.39 | 11.1 | 11.5 |
| *ε*_shoulder_/*ε*_Soret_ | 0.341 | 0.366 | 0.381 | 0.424 | 0.453 |
| $\varepsilon_{\mathrm{Por}}(\lambda) = \Sigma\{A_{i}\times exp(-{(\lambda-\mu_{i})}^{2}/2\sigma_{i}^{2})\}$  $\varepsilon_{\mathrm{shoulder}}/\varepsilon_{\mathrm{Soret}}=A_{2}/A_{1}$ | | | | | |

**Table S19.** Rejection test (95.4% confidence interval) for the *Φ*_F_ of **1a**–**c@B** and **1a**–**c@C**

| Entry | *Φ*_F_ on AuNP [%] | | | | | |
| --- | --- | --- | --- | --- | --- | --- |
|  | **1a@B** | **1b@B** | **1c@B** | **1a@C** | **1b@C** | **1c@C** |
| 1 | 0.113 | 0.138 | 0.268 | 0.186 | 0.173 | 0.218 |
| 2 | 0.178 | 0.100 | 0.195 | 0.179 | 0.132 | 0.176 |
| 3 | 0.094 | 0.150 | 0.199 | 0.076 | 0.171 | 0.164 |
| 4 | 0.065 | 0.131 | 0.202 | 0.116 | 0.140 | 0.183 |
| 5 | 0.087 | 0.153 | 0.143 | 0.118 | 0.149 | 0.183 |
| average | 0.107 | 0.134 | 0.201 | 0.135 | 0.153 | 0.185 |
| 2SD | 0.077 | 0.038 | 0.080 | 0.083 | 0.033 | 0.036 |
| ■ Rejected  ■ Not rejected | | | | | | |

**References**

Imahori, H., Norieda, H., Nishimura, Y., Yamazaki, I., Higuchi, K., Kato, N., et al. (2000). Chain Length Effect on the Structure and Photoelectrochemical Properties of Self-Assembled Monolayers of Porphyrins on Gold Electrodes. *J. Phys. Chem. B* 104, 1253–1260. doi:10.1021/jp992768f.

Shinohara, A., and Shinmori, H. (2016). Controlled Generation of Singlet Oxygen by Porphyrin-Appended Gold Nanoparticles. *Bull. Chem. Soc. Jpn.* 89, 1341–1343. doi:10.1246/bcsj.20160254.
